# Antiglucocorticoid RU38486 reduces net protein catabolism in experimental acute renal failure

**DOI:** 10.1186/1471-2369-6-2

**Published:** 2005-02-17

**Authors:** Adrian Mondry

**Affiliations:** 1 Bioinformatics Institute, 30 Biopolis Street, #07-01 Matrix Building, 138671 Singapore

## Abstract

**Background:**

In acute renal failure, a pronounced net protein catabolism occurs that has long been associated with corticoid action. By competitively blocking the glucocorticoid receptor with the potent antiglucocorticoid RU 38486, the present study addressed the question to what extent does corticoid action specific to uremia cause the observed muscle degradation, and does inhibition of glucocorticoid action reduce the protein wasting?

**Methods:**

RU 38486 was administered in a dose of 50 mg/kg/24 h for 48 h after operation to fasted bilaterally nephrectomized (BNX) male adult Wistar rats and sham operated (SHAM) controls. Protein turnover was evaluated by high performance liquid chromatography (HPLC) of amino acid efflux in sera from isolated perfused hindquarters of animals treated with RU 38486 versus untreated controls.

**Results:**

Administration of RU 38486 reduces the total amino acid efflux (TAAE) by 18.6% in SHAM and 15.6% in BNX and efflux of the indicator of net protein turnover, phenylalanine (Phe) by 33.3% in SHAM and 13% in BNX animals as compared to the equally operated, but untreated animals. However, the significantly higher protein degradation observed in BNX (0.6 ± 0.2 nmol/min/g muscle) versus SHAM (0.2 ± 0.1 nmol/min/g muscle) rats, as demonstrated by the marker of myofribrillar proteolytic rate, 3-Methylhistidine (3 MH) remains unaffected by administration of RU 38486 (0.5 ± 0.1 v. 0.2 ± 0.1 nmol/min/g muscle in BNX v. SHAM).

**Conclusion:**

RU 38486 does not act on changes of muscular protein turnover specific to uremia but reduces the effect of stress- stimulated elevated corticosterone secretion arising from surgery and fasting. A potentially beneficial effect against stress- induced catabolism in severe illness can be postulated that merits further study.

## Background

As part of the complex uremic metabolic syndrome, pronounced disturbances of carbohydrate and lipid metabolism are commonly observed, as are pathologic changes of amino acid and protein turnover [1]. An increased net protein degradation in uremia was seen as early as 1949 by *Persike and Addis*[2], and in the same year, *Bondy et al*. [3] showed that adrenal hormones are involved therein. These early findings were validated in the late eighties by *Schäfer et al*. [4,5] who postulated a leading role for glucocorticoids as cause of the observed changes. *Schäfer et. al *used the experimental approach of inhibiting activation of the glucocorticoid receptor by enteral application of the potent glucocorticoid antagonist RU 38486 in acutely uremic rats [6] and found a decrease both in the accumulation of 3-methylhistidine, an amino acid that is produced in actomyosine catabolism and is not further metabolized, and in the activity of myofibrillar protease. However, while an effect of RU38486 on liver gluconeogenesis and urea synthesis in uremia could be demonstrated [7,8], so far there is no proof of a direct action of RU38486 on muscle metabolism in uremia. To address this problem, the present study made use of the classical experimental design of the isolated perfused hindquarter of the rat [9], in which roughly 40% of the rat body's total muscle mass can be evaluated under closely defined in vitro conditions.

With this experimental design and by comparing sham-operated and bilaterally nephrectomized animals, the present study looked at the question to what extent does corticoid action specific to uremia cause the observed muscle degradation, and does inhibition of glucocorticoid action reduce the protein wasting?

## Methods

Animal experimentation was carried out on male Wistar rats, aged 11–15 weeks, weighing 217–225 g, from the animal experimentation facilities of Heinrich Heine University, Düsseldorf. Permission to use animals for experimentation was given by Regierungspräsident Düsseldorf, file nr. 26.4203.1-217/87 according to German federal law. Surgery for nephrectomy, sham nephrectomy and preparation for perfusion was carried out under narcosis with hexobarbital (EVIPAN- Na: 15–20 mg/ 100 g BW).

Bilateral nephrectomy was performed using a dorsal access, ligation of renal vessel string, and excision of the kidney, leaving the adrenal glands in place. Sham operated animals underwent the same manipulations except for the ligation and excision. After surgery, animals were fasted for 48 h until perfusion. Nephrectomized animals had access to drinking water on the day of surgery for 8 hours, then were deprived of liquid to avoid lung edema. Sham animals had free access to drinking water throughout.

Animals were randomly assigned to one of four groups: bilaterally nephrectomized (BNX) and sham operated (SHAM) treated with RU38486 and untreated BNX and SHAM animals. For treatment, RU38486 was dissolved in phenylmethanol, then mixed with sesame oil to form a milky suspension which was injected into subcutaneously into the lateral abdomen in three subdoses within 24 h, adding up to a total dose of 5 mg/100 g/BW.

48 h after initial surgery, animals were narcotized and prepared for perfusion as previously described[10].

The hindquarter was linked to the recirculation system after full passage of 70 ml of pre- perfusion medium, as shown in illustration 1. The pre- perfusion medium was discarded and not used for the recirculation experiment.

The perfusion was carried out with a half- synthetic medium on the basis of Krebs- Ringer- bicarbonate buffer (KRB), pH 7.38 [11]. Oxygen carriers were calf erythrocytes prepared from fresh calf blood sampled two days before experimentation and maintained with 300 mg/l Ampicillin and 220 ml/l citric acid/ glucose stabilizer. Bovine albumine maintained the physiologically correct oncotic pressure. 10^-6 ^mmol/l phentolamine were added to avoid vessel contractions.

During perfusion, the arterial pH and perfusate oxygenation were monitored using a pH- meter and a total oxygen content analyzer (LEX- O2- CON, Lexington Instr., Mass., USA). At the beginning and end of perfusion, plasma samples were frozen for amino acid analysis. Amino acid analysis by HPLC was carried out using 25 μl of deproteinized perfusate sample, mixed with o-phthaldialdehyde (OPA)/3-mercaptopropionic acid to form OPA- adducts that were separated on a reversed phase column and measured by fluometry. Quantification was done by comparison with a standard amino acid mix including 3-methylhistidine. Of the 20 proteinogenic amino acids, cysteine, proline, and asparic acid were not included in HPLC analysis.

Statistical analysis was done using the "Student" t- test for ungrouped, non- paired data with f = n1 + n2-2 and a significance level of p < 0.05.

## Results

### Loss of body weight (BW)

During the 48 h fasting period between operation and perfusion, animals had a pronounced loss of BW (table 1). In SHAM, it was 35.5 ± 5.3 g; administration of RU 38486 reduced this to 27.6 ± 5.9 g (p < 0.05). Nephrectomized animals demonstrated a much less pronounced loss of BW due to a significant increase in tissue hydratation (Table 2). RU 38486 reduces the weight loss in nephrectomized animals, too; however, this effect is much less pronounced and lacks statistical significance (BNX 15.7 ± 4.8 g, BNX + RU 12.2 ± 4.8 g).

### Oxygen utilization and development of acidosis

Oxygen utilization in the perfused muscle tissues is roughly the same in all four groups (data not shown) and equal to in vivo data previously reported [12] from rats after 24 hour fasting. As expected, pH dropped significantly lower during perfusion in the nephrectomized groups (SHAM: 7.378 ± 0.033, BNX 7.321 ± 0.018, p = <0.01; SHAM + RU 7.4 ± 0.031, BNX + RU 7.312 ± 0.013, p < 0.001).

### Parameters of amino acid and protein metabolism

*Total amount and spectrum of amino acids released during reperfusion: *During perfusion, amino acids are released in varying amounts as shown in ill. 2. Nephrectomized animals (BNX) showed a general increase in amino acid release. This, however, is significant only in a few individual amino acids. The total amino acid efflux increases by 10,4 % (p = 0.05) without qualitative change. Notable exception is glycine, which is released to a lesser amount in nephrectomized animals. *Amino acid release after treatment with RU 38486: *Nephrectomy equally increases the amino acid efflux in animals treated with RU 38486 by 14.4% (p < 0.05) without change in spectrum. The total efflux of amino acids, however, is significantly reduced in the comparison SHAM/ SHAM+RU (-18.6%, p < 0.05) and BNX/ BNX+RU (-15.6%, p < 0.001). *Release of 3-methylhistidine: *3-methylhistidine, a derivate of histidin mainly from actin and myosin in sceletal muscle and intestinal mucosa [13], is not reutilized after proteolysis, but excreted via the kidney as 3-methylhistidine or N- acetyl- 3-methylhistidine. During reperfusion of sham- nephrectomized animals, 3-methylhistidine is released from the hindquarter to a small amount that is incresed by roughly 300% in the nephrectomized animals. Administration of RU38486 has no effect on 3-methylhistidine efflux. (table 3).

## Discussion

### Background

Acute renal failure is a catabolic state, and unfortunately the inherent acceleration of protein breakdown cannot be suppressed effectively by provision of exogenous nutritional substrates [14]. The situation is multicausal. Unspecific mechanisms induced by the process of acute disease, underlying illness and associated complications are just one side of the problem. On the other, one observes specific uremic effects, insulin resistance, hormonal derangements, metabolic acidosis, circulating proteases and other inflammatory mediators together with effects induced by the acute loss of renal function and the type and intensity of renal replacement therapy [1,15-17].

One factor that has for long been associated with the disturbances of protein metabolism is glucocorticoid action. The first observations date back to the nineteen-forties, when Persike and Addis [2] reported an increased urea- nitrogen production in experimental uremia, and Bondy and coworkers [3] demonstrated that adrenal hormones were involved in this dysregulation. Half a century later, it is still not fully understood to what extent steroid hormone action is responsible for the catabolic situation observed in renal insufficiency[18]. It has been shown that administration of high doses of glucocorticoids to adrenalectomized rats resulted in decreased protein synthesis, increased protein degradation, and a negative nitrogen balance [19]. In patients with chronic renal failure, a positive correlation between muscle proteolysis and the plasma cortisol level has been observed [20]. The in vivo influence of both glucocorticoids and metabolic acidosis on muscle proteolysis has been elucidated in whole- body leucine turnover studies in adrenalectomized rats [21]. These findings indicate that glucocorticoids play an important role in net protein degradation. Price formulated this so: "glucocorticoids are required but not directly responsible for the acidosis-induced increase in the mRNAs encoding proteins of this degradative pathway"[22].

### Experimental approach

In order to evaluate the relative importance of glucocorticoid action on protein metabolism in acute renal failure, an experimental setting was chosen that allowed to study glucocorticoid action indirectly by selective blockade of the glucocorticoid receptor with the potent antiglucocorticoid RU38486, a substance that binds to the receptor without activating the further process of transcription [23]. Parenteral administration of a total of 50 mg/kg BW/ d of RU38486 allowed to avoid additional irritation of the animals by a gastric catheter in the postoperative phase. Relevant action of RU38486 has been observed in enteral substitution at a dose of 20 mg/kg BW/d [6]. The degree to which RU 38486 blocks the glucocorticoid receptor depends very much on the mode of application, and the target tissue. While a recent study[24] shows that 80% of glucocorticoid receptors are blocked in rat muscle following oral application of mifepristone of 50 mg/ kg BW, Kim et al.[25] demonstrated effective blocking of glucocorticoid receptors in rat brain following subcutaneous application of 80 mg/ kg over two days. Schaefer et al.[6], on whose experimental set- up the present study was modeled, had reported significant effects of an oral dose of 20 mg/ kg on muscle. In view of this, the choice for the experimental procedure seems justified as the present study uses a substantially higher dose.

The isolated perfusion procedure introduced by Ruderman[9] is well established for the representative study of muscle metabolism. In this setting, the perfused muscle mass is approximately 40% of the total muscle mass. Taking into consideration the different metabolic requirements of the perfused tissues, roughly 90% of oxydative metabolism occurs in the muscle[12], making this experimental setting truly a skeletal muscle preparation that permits the observation of even very discrete metabolic changes during reperfusion.

In the given experimental setting, it is difficult to account for the in detail contribution of protein degradation, amino acid intermediate metabolism, and protein synthesis. Factors that modify the efflux are transport systems in the cell membrane [26-28], which can be concentration- dependent (system L) or acting against the concentration gradient (system A), and the intermediary metabolism within the muscle cells[29]. Numerous previous assessments of the metabolic situation in the isolated perfused hindlimb demonstrate that these factors are relatively minor contributors, while amino acid efflux is nearly exclusively characterized by the net balance of protein metabolism both in anabolic and catabolic situations[12,30-34]. It is mostly due to changes in skeletal muscle, with only minor contributions from other tissues in this preparation [35-37].

### Results

Sham- operated animals are catabolic at the time of perfusion, having lost about 36 g BW (see results), which is approximately 16% of initial BW. Rats of this age are still growing, with an increase of approx. 5 g/ day (2–3% BW)[38]. The weight loss is due to both lipolysis[39] and protein loss[40], which causes the typical increase of amino acid release in the hindquarter of fasting rats[12]. Compared to data[34] from non- operated rats fasted for 48 hours under otherwise identical conditions, the total amino acid release is increased by 30% in the sham- operated rats described here. While nephrectomy increases the amino acid release by approximately 15%, the relative decrease of amino acid release following administration of RU 38486 is similar in both nephrectomized and sham- operated animals. This indicates a stress- accentuated adaptation to fasting caused by corticosteron secretion[41] increased beyond the normal range, with a further effect of uremia. Increased amino acid serum concentrations during fasting are mostly due to an inhibition of protein synthesis[42], although proteolysis mainly of myofibrillary proteins does play a role[43]. However, corticosterone is only one of several effectors at play. RU 38486 affects neither acidosis nor lactate/ pyruvate ratio. Both factors may contribute to the continuously increased amino acid release. In the case of acidosis, this may be due to an action on acid inhibitable transporters such as system A which reduce the supply of nutrients to the cells[28]. Another possible mechanism is through inhibition of leptin by acidosis[44], which in neutral pH might counteract muscle wasting[45]. Balancing acidosis in chronically uremic rats with increased corticosterone secretion inhibited protein degradation, but had no effect on the defective protein synthesis[21]. More recently, RU 38486 was shown to be ineffective in blocking acid- mediated protein degradation as its action is only an indirect one, mediated via insuline- like growth factor I (IGF- I)[24,46]. These findings indicate that RU 38486 acts through an inhibition of the corticosterone- mediated decrease of protein- synthesis without affecting other factors that act predominantly on the level of protein degradation. While all these and more factors contribute to the muscle degradation seen in excess glucocorticoid situations, the mechanisms responsible *in ultima causa *remain still unclear[18]. Nephrectomy enhances the catabolic situation: the total amino acid efflux is increased by roughly 15% compared to sham- operated animals. The relative increase of amino acids that are not metabolized, such as phenylalanin and tyrosin, indicates that this effect is due to the acute and complex metabolic situation of uremia, without differenciating between inhibition of protein synthesis and stimulation of protein degradation. A multitude of effectors partake in this metabolic turmoil [15], of which glucocorticoids have been accused of playing a leading role [47]. At first sight, this opinion is supported by the finding that isolated hindquarters of animals treated with RU 38486 show a significant reduction of total amino acid efflux (16–19%, p < o.05- p <0.001) compared to untreated animals that underwent the same surgical procedure, indicating that RU38486 inhibits some common degrading influence on protein metabolism.

By contrast, in the comparison of the two groups treated with RU38486, the amino acid release remains increased in nephrectomized animals, albeit to a lesser extent, stressing the very point that glucocorticoids are only one of several factors that contribute to the net protein wasting. Chronically uremic rats with increased corticosterone- secretion [48] showed a less pronounced increase in protein degradation when acidosis was balanced while the defective protein synthesis remained unchanged. In the present experimental setting, acidosis evolving during perfusion was not corrected for. Acidosis and glucocorticoid action are seen as concomitant factors in the activation of the ubiquitin- proteasome pathway of muscle proteolysis [49], and a pH- responsive element in the promoter region for the ubiquitin- proteasome pathway has been reported [47]. In the situation of uncorrected acidosis, it therefore seems likely RU 38486 may have had an inhibitory effect on the corticoid- induced decrease in protein synthesis without influencing the proteolytogenic effects of other putative agents.

This presumption is supported by the finding that RU 38486 had no effect on the efflux of 3-methylhistidin. While this result is in contrast to Schäfer et al. [6,8], *Lowell et al*. found no reduction of the efflux of 3-methylhistidin after adrenalectomy in the perfused hindquarter of fasted animals [50], and in rats with chronic uremia, RU38486- resistant protein catabolism with unchanged release of 3-methylhistidin has been demonstrated in vivo by *Teschner *[51]. As responsiveness of protein synthesis and degradation to amino acid availability seem to be regulated differentially [52] and activation of glucocorticoid- mediated proteolysis occurs only at relatively elevated hormone levels [53] compared to the inhibition of protein synthesis [54], it seems possible to speculate that RU38486 may have a more pronounced effect on net protein catabolism at substantially higher doses.

The presented data fail to show that RU38486 inhibits glucocorticoid action in the specific uremic setting while it clearly reduces the elevated net protein catabolism compared to non- operated animals. This suggests that glucocorticoid mediated protein wasting in acute uremia is rather a by- product of the overall stress, in the present experimental setting caused by surgery and fasting, than due to an independent action specific to uremia.

While this finding abolishes hopes to counteract muscle wasting in uremia by administration of an anticorticoid drug and indirectly rather stresses the well described[55] clinical importance of a balanced acid- base status, it may open speculation about the usefulness of RU486 in post- traumatic states and severe illness.

## Conclusion

Both sham- operated and nephrectomized animals show an increase in net protein catabolism. RU38486 clearly reduces the net protein wasting in both groups, but the increase in net protein catabolism observed over sham- operated animals remains unchanged in nephrectomized rats. The effect of antiglucocorticoid RU38486 may be attributed to an inhibition of fasting and operative stress- induced cortisol action which, even when within the physiological range, promotes increased protein turnover [56], and to a protective effect against the inhibition of protein synthesis. While RU38486 had no effect on net protein catabolism that could be specifically attributed to uremia, the demonstrated anticatabolic effectiveness in a stress accentuated metabolic situation should be studied more closely. Possible targets for therapeutic application under this aspect include post- traumatic states and severe illness.

## Competing interests

The author(s) declare that they have no competing interests.

## Authors' contributions

A.M. carried out animal experimentation, sample analysis, statistical analysis and wrote the manuscript.

## Pre-publication history

The pre-publication history for this paper can be accessed here:



## Figures and Tables

**Figure 1:**
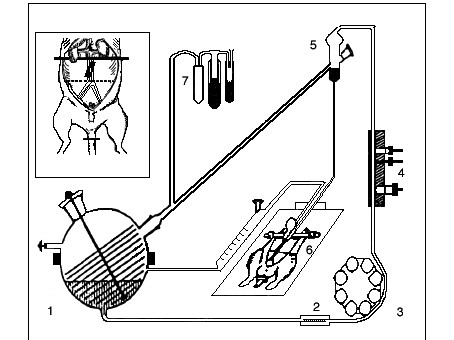
**A: **Isolated rat hindquarter preparation. The perfused muscle mass is below the dotted line. The tail is clamped off. **B: **Recirculating perfusion system. Heart piece is the oxygenator (1), wherein the medium is gassed (95% O_2_, 5% CO_2_) and from where it is pumped (3) up via a filter (nylon mash, 20 μm width) (2) and pH and pO_2 _probes (4) to attain hydrostatic pressure of 80 mm Hg (5) before entering the hindquarter (6). Intermediate sampling and pH adjustment can be carried out if necessary (7).

**Figure 2:**
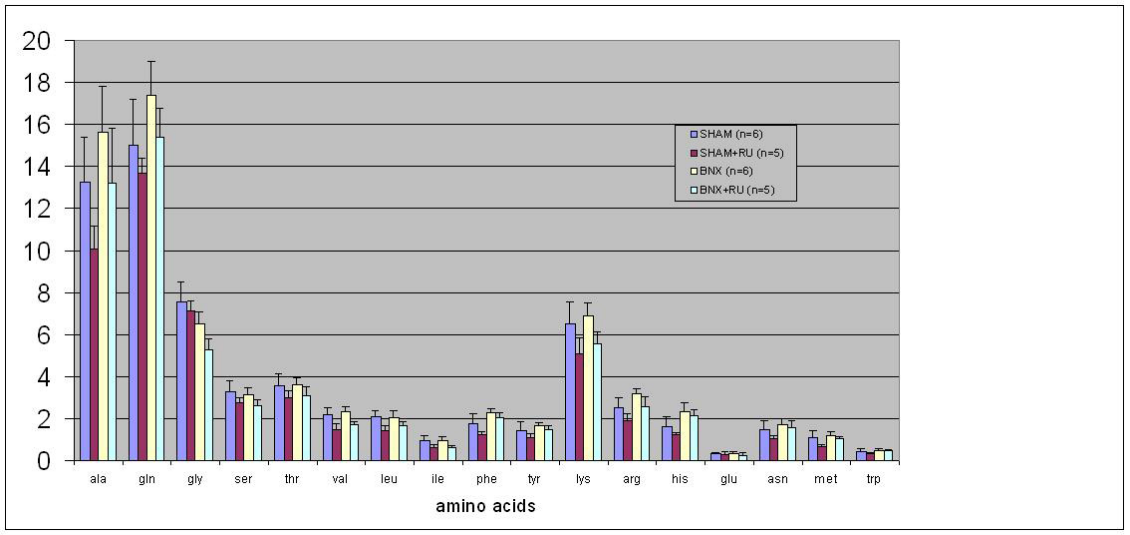
Release rate of amino acids (nmol/ min/ g muscle) from isolated perfused rat hindquarter. Error bars indicate standard deviation. The figure is based on tables 4 and 5 which give the p-values for the comparison between sham- operated and nephrectomized animals (table 4) and RU 38486 untreated and treated groups (table 5), respectively.

**Table 1:** Body weight (BW) at day of surgical intervention (OP) and perfusion (EX). Values are gram ± standard deviation. No significant intergroup differences.

	BW [g] at OP	BW [g] at EX	Weight loss [g]
SHAM (n = 6)	223.67 ± 8.02	188.17 ± 7.28	35.5 ± 5.3
BNX (n = 6)	217.33 ± 13.81	201.67 ± 11.91	15.7 ± 4.8
SHAM + RU (n = 5)	223.60 ± 9.21	196.00 ± 9.70	27.6 ± 5.9
BNX + RU (n = 5)	225.20 ± 5.36	213.00 ± 7.35	12.2 ± 4.8

**Table 2:** Dry weight (DW) in % of total tissue weight and hydration (Hy) at day of perfusion in ml/ g of total tissue weight. Average values ± standard deviation. Intergroup differences with significance levels of: *p < 0,001; #p < 0,05.

	SHAM*	BNX*	SHAM + RU#	BNX + RU#
n	6	6	5	5
DW [%]	24.41 ± 1.46	21.07 ± 0.98	24.26 ± 1.14	22.10 ± 1.46
Hy [ml/g]	0.761 ± 0.015	0.795 ± 0.010	0.762 ± 0.012	0.784 ± 0.015

**Table 3:** Total amino acid efflux (TAAE) and release of 3-methyl histidine (3 MH) in serum from isolated perfused rat hindquarter at 60 min. perfusion. Values given in nmol/min/g muscle + standard-deviation. Intergroup differences with significance of: #p < 0,01; *p < 0,005; ~, $p < 0,001.

	SHAM	BNX	SHAM + RU	BNX + RU
n	6	6	5	5
TAAE	65.05 ± 5.88*	71.83 ± 5.02#	52.98 ± 2.96*	60.61 ± 5.62#
3 MH	0.2 ± 0.09~	0.55 ± 0.15~	0.18 ± 0.06$	0.53 ± 0.07$

**Table 4:** Amino acid release from the isolated perfused hindlimb. P- values indicate significant differences in the comparison between sham- operated and nephrectomized animals, either without or with RU 38486 treatment. A: p- value for SHAM vs. SHAM + RU; B: p- value for BNX vs. BNX + RU; C: p- value for SHAM vs. BNX; D: p- value for SHAM + RU vs. BNX + RU.

	SHAM	SHAM+RU	BNX	BNX+RU	A: p-value	B: p-value	C: p-value	D: p-value
Ala	13.26	10.08	15.61	13.23	<0.02	n.s	<0.05	<0.05
Glu	15	13.69	17.39	15.37	n.s	n.s	<0.05	<0.05
Gly	7.56	7.11	6.53	5.26	n.s	<0.005	n.s	<0.001
Ser	3.27	2.75	3.12	2.6	n.s	<0.025	n.s	n.s
Thr	3.58	2.98	3.62	3.11	n.s	~0.05	n.s	n.s
Val	2.2	1.49	2.32	1.7	<0.005	<0.001	n.s	n.s
Leu	2.08	1.43	2.04	1.66	<0.005	~0.05	n.s	n.s
Ile	0.95	0.63	0.97	0.63	<0.025	<0.005	n.s	n.s
Phe	1.78	1.23	2.29	2.02	<0.05	n.s	<0.05	<0.001
Tyr	1.41	1.09	1.68	1.48	n.s	~0.05	n.s	<0.01
Lys	6.53	5.08	6.91	5.54	<0.05	<0.005	n.s	n.s
Arg	2.5	1.9	3.16	2.57	<0.05	<0.05	<0.02	<0.05
His	1.63	1.22	2.33	2.12	n.s	n.s	<0.025	<0.001
Glu	0.31	0.28	0.32	0.26	n.s	n.s	n.s	n.s
Asn	1.49	1.05	1.7	1.57	n.s	n.s	n.s	<0.02
Met	1.1	0.65	1.17	1.03	<0.02	n.s	n.s	<0.001
Trp	0.43	0.32	0.47	0.46	n.s	n.s	n.s	<0.01
